# Integrative approaches to healthy aging in postmenopausal women: the synergistic benefits of exercise combined with functional foods and dietary supplements

**DOI:** 10.3389/fendo.2026.1717915

**Published:** 2026-03-20

**Authors:** Hongli Fu, Xiaowei Lei

**Affiliations:** 1 Department of Physical Education, Luoyang Institute of Science and Technology, Luoyang, Henan, China; 2 Sports Department, Chang’an University, Xi’an, Shaanxi, China

**Keywords:** bone health, exercise, polyphenol, postmenopausal women, quality of life

## Abstract

The transition into menopause is accompanied by physiological changes that can impact women’s health and quality of life (QoL), including impaired bone metabolism, metabolic dysregulation, and diminished mental well-being. This review explores integrative, non-pharmacological strategies—specifically structured exercise training combined with functional foods and dietary supplements—as synergistic approaches to mitigate these challenges. We synthesize current evidence on the endocrine, skeletal, metabolic, and psychological effects of pairing physical training with bioactive components, specifically phytoestrogens, polyphenols, omega-3 fatty acids, and vitamin D. Special emphasis is placed on the interaction between these interventions and hormonal regulation, bone turnover, and inflammatory pathways. While preclinical models suggest potent mechanistic synergies, clinical evidence remains heterogeneous. Collectively, the data suggests that tailored lifestyle interventions may offer promising strategies to enhance bone health and QoL. However, variability in study designs and dosages currently limits the ability to prescribe standardized protocols. Future research must focus on large-scale, randomized trials to establish optimal combinatorial dosages.

## 1 Introduction

The menopausal transition represents a critical biological juncture characterized by the cessation of ovarian function and a precipitous decline in endogenous estrogen and progesterone (1–3). This hormonal withdrawal precipitates a cascade of systemic physiological alterations, including endothelial dysfunction, accelerated bone resorption outpacing formation, and metabolic dysregulation, collectively increasing the risk of cardiovascular disease, osteoporosis, and metabolic syndrome (4–8). While Hormone Replacement Therapy (HRT) remains the clinical gold standard for managing vasomotor symptoms and preserving bone density, its use is contraindicated in certain populations and remains a subject of caution for others due to potential risks of venous thromboembolism and specific malignancies (9, 10). Consequently, there is a growing imperative to identify effective non-pharmacological interventions that can mimic the protective effects of estrogen without the associated adverse events.

**Figure 1:**
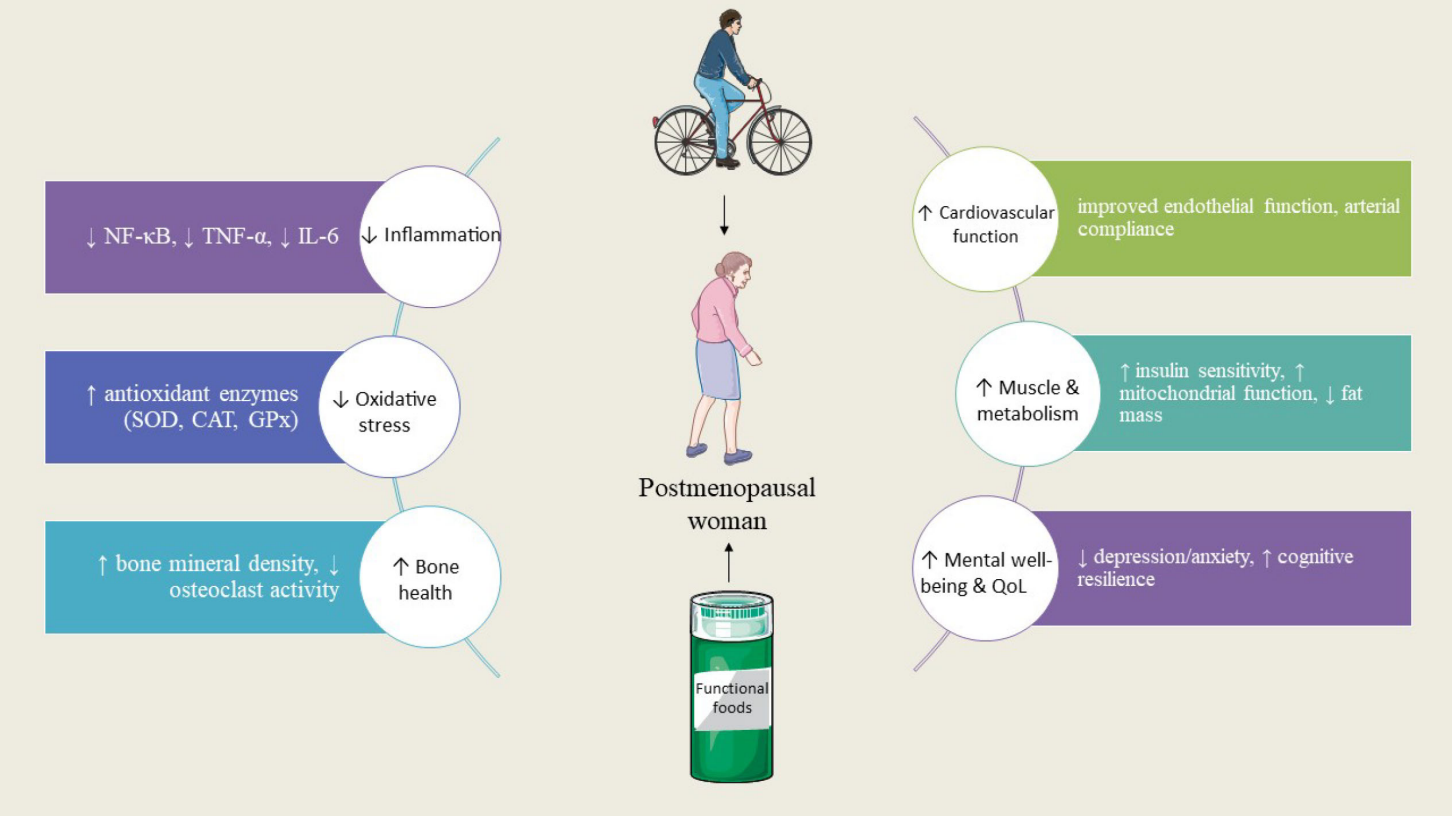
Overview of how exercise and functional foods act synergistically in postmenopausal women. Both interventions converge on shared mechanisms, including reduced inflammation and oxidative stress, improved bone and muscle function, enhanced cardiovascular health, and better psychological well-being, ultimately promoting healthy aging and quality of life.

Lifestyle modifications, specifically structured exercise training and dietary interventions, have emerged as potent frontline strategies. However, while the individual benefits of structured exercise training (encompassing resistance, aerobic, and high-intensity interval modalities) and functional food supplementation are well-documented, their combined therapeutic potential remains underutilized (11, 12). Exercise acts as a powerful physiological stressor that stimulates osteogenesis and metabolic flexibility, while bioactive compounds in functional foods—such as polyphenols and phytoestrogens—modulate oxidative stress and inflammatory signaling pathways (13–15). Theoretically, integrating these distinct mechanisms could yield a synergistic effect, offering a more comprehensive defense against the multisystem decline associated with menopause. This synergism is physiologically significant because exercise primarily delivers mechanical and metabolic stimuli essential for tissue adaptation, whereas functional foods amplify intracellular signaling cascades, optimize redox homeostasis, and enhance tissue sensitivity to these stimuli (16). Together, they partially emulate the protective roles of estrogen-mediated pathways, which are diminished post-menopause, thereby collectively bolstering cardiometabolic resilience and musculoskeletal integrity (17, 18). Polyphenols such as turmeric, green tea catechins, soy isoflavones, and ginger have been identified for their potent antioxidant and anti-inflammatory properties (19–22). Flavonoids possess strong antioxidant properties, which play a significant role in many of the effects that will be addressed. Being single-electron contributors, they possess the ability to counteract the effects of free radicals and eliminate harmful reactive oxygen species. This, in turn, helps to hinder the oxidation of unsaturated fatty acids in membranes (23, 24). The immune system has been proven to be an effective measure of overall well-being, rate of ageing, and length of survival (25, 26). One of estrogen’s primary roles in aging is its regulation of the immune system (27, 28). Estrogen has immunomodulatory effects, enhancing the body’s ability to fight infections and manage inflammation (27, 29, 30). It helps maintain a balance between pro-inflammatory and anti-inflammatory responses, ensuring the immune system functions optimally (29, 31, 32). During menopause, the drop in estrogen levels can lead to a dysregulated immune response, increasing the susceptibility to autoimmune diseases, infections, and inflammatory conditions (33). This decline may also contribute to the accelerated aging process seen in postmenopausal women, characterized by weakened immune defenses and greater vulnerability to age-related diseases like osteoporosis, cardiovascular disease, and cognitive decline (33). Moreover, preserving wellness serves as the main foundation for a well-operating duration of one’s life and is impacted by around 25% from inherited traits and 75% from individual decisions and surroundings. The addition of ample antioxidants to the dietary intake of senior citizens results in an improvement in various immune cell functions, a decrease in oxidative stress, and ultimately an increase in lifespan (34, 35). Despite an expanding corpus of scholarly work that examines the distinct advantages of functional food intake and physical exercise in postmenopausal women, a notable deficiency persists in comprehensive reviews that amalgamate both interventions while concentrating on their synergistic impacts on physical and mental well-being, metabolic profiles, and overall quality of life (QoL). The majority of extant reviews exhibit a narrow focus—generally analyzing either nutritional aspects or physical activity independently, without delving into their interactive mechanisms or collective therapeutic efficacy within the postmenopausal demographic. Furthermore, the metabolic challenges and QoL concerns that are specific to postmenopausal women, including hormonal imbalances, elevated cardiometabolic risk, cognitive deterioration, and mood disorders, necessitate a holistic and integrative approach. To date, there has been no review that has cohesively synthesized contemporary findings from clinical, biochemical, and physiological perspectives in a comprehensive manner that elucidates how functional foods and structured exercise collectively influence endocrine regulation, inflammatory pathways, metabolic adaptation, and psychological resilience throughout the postmenopausal transition. This scholarly review addresses a significant deficiency in the current literature by presenting a comprehensive and mechanistically informed discourse, underscoring the interplay between nutritional and Exercise interventions. It furnishes healthcare practitioners, academic researchers, and policymakers with a contemporary, evidence-based framework designed to facilitate the formulation of individualized lifestyle strategies aimed at enhancing long-term health outcomes in postmenopausal women. In light of the global demographic transition toward aging populations, this pertinent review offers essential insights for both preventive healthcare and precision nutrition within the context of women’s health (**Figure 1**).

While a broad spectrum of functional foods and bioactive compounds such as cocoa polyphenols, dietary nitrates, and various phytoestrogens, hold promise for postmenopausal health, this review specifically prioritizes compounds with established or emerging evidence of synergistic efficacy when combined with physical exercise. We focused on four distinct categories: (1) Polyphenols (Curcumin, Green Tea, Ginger): Selected for their potent anti-inflammatory and antioxidant properties that potentially amplify exercise-induced adaptations; (2) Phytoestrogens (Soy Isoflavones): Included due to their structural similarity to estrogen and potential to mitigate bone loss and metabolic dysregulation in the absence of endogenous hormones; (3) Micronutrients (Vitamin D): Essential for musculoskeletal integrity and often synergistic with resistance training; and (4) Omega-3 Fatty Acids: Critical for cardiovascular and cognitive health, with evidence suggesting enhanced benefits when paired with aerobic exercise. This targeted selection allows for a deeper analysis of specific nutrient-exercise interactions rather than a superficial overview of all available functional foods.

While previous reviews have often examined nutritional or physical interventions in isolation, the systemic nature of the menopausal transition characterized by simultaneous declines in endocrine, skeletal, and metabolic health, necessitates a holistic, multi-modal approach. Consequently, this review specifically targets the synergistic potential of exercise combined with functional foods. The scope is intentionally comprehensive to encompass the inter-related physiological systems most affected by estrogen withdrawal. We critically synthesize evidence regarding interventions that target three key domains: (1) inflammatory and oxidative pathways (targeted by polyphenols and omega-3s), (2) musculoskeletal integrity (targeted by vitamin D/calcium and resistance training), and (3) metabolic regulation. By integrating these specific functional foods with varied exercise modalities, we aim to move beyond descriptive summaries to evaluate how combinatorial strategies can effectively mitigate the multifaceted risks of postmenopausal aging. This review identifies research gaps and suggests optimized combinatorial strategies for this demographic.

## 2 Search strategy and selection criteria

To ensure a comprehensive synthesis of the literature, we conducted an electronic search of the PubMed, Scopus, and Google Scholar databases for studies published up to December 2025. The search strategy employed Boolean operators to combine terms related to the population (“postmenopausal women,” “menopause,” “ovariectomized models”), interventions (“exercise,” “resistance training,” “aerobic training,” “functional foods,” “polyphenols,” “phytoestrogens,” “vitamin D,” “omega-3,” “curcumin,” “green tea,” “soy,” “ginger”), and outcomes (“bone mineral density,” “inflammation,” “oxidative stress,” “quality of life,” “metabolic profile”).

We prioritized randomized controlled trials (RCTs) and longitudinal studies involving postmenopausal women. Preclinical studies (animal models) were included specifically to elucidate molecular mechanisms not observable in human trials (e.g., histological bone changes or tissue-specific gene expression). Studies were excluded if they: (1) were not published in English; (2) focused exclusively on hormone replacement therapy (HRT) without lifestyle arms; or (3) utilized non-standardized extracts without clear dosage information. This selection process aimed to filter for high-quality evidence regarding the synergistic efficacy of combined lifestyle interventions.

## 3 Operational definitions

To ensure clarity and consistency, the following operational definitions are applied throughout this review:

- Physical activity vs. exercise training: We distinguish between physical activity (any bodily movement resulting in energy expenditure) and exercise training (planned, structured, and repetitive physical activity aimed at improving physical fitness). The review focuses primarily on the latter.
- Functional foods vs. dietary supplements: Functional foods are defined as whole or fortified foods that provide health benefits beyond basic nutrition (e.g., soy, green tea). Dietary supplements refer to isolated bioactive compounds or micronutrients administered in non-food matrices (e.g., Vitamin D capsules, curcumin extracts).
- Synergy: In this context, synergy refers to an interaction where the combined effect of exercise and a dietary intervention exceeds the sum of their individual effects.

## 4 Synergies targeting inflammation and oxidative stress

### 4.1 The beneficial effects of exercise and turmeric intake in postmenopausal women

**Figure 2:**
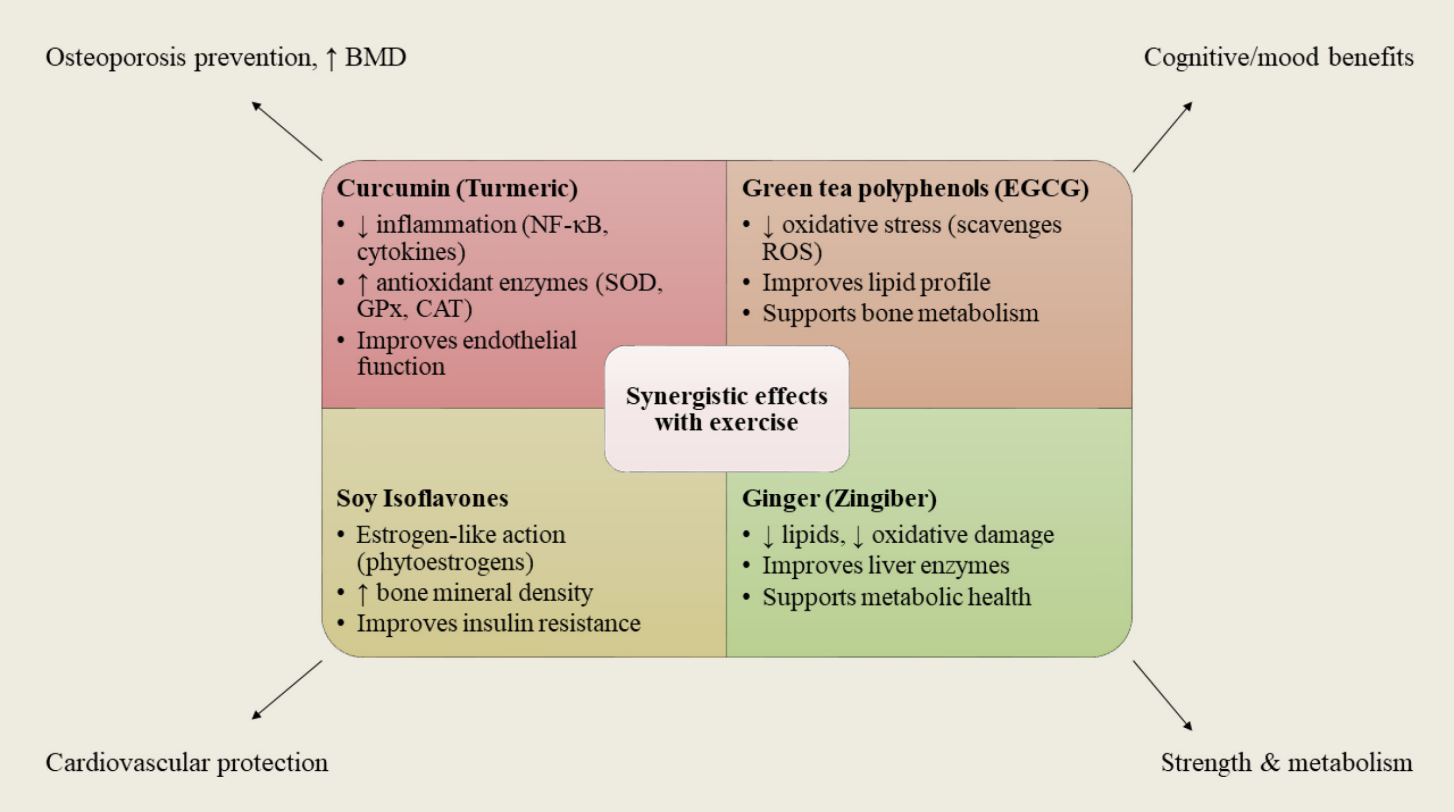
Synergistic actions of polyphenol-rich foods (curcumin, green tea, soy, ginger) combined with exercise. Each functional food contributes unique molecular and metabolic benefits, but all converge with exercise on shared outcomes such as stronger bones, improved cardiovascular function, better metabolism, and enhanced mental well-being.

New studies have demonstrated that oxidative stress and inflammation play a significant role in the development of menopausal symptoms, such as hot flashes and night sweats. Studies have shown that curcumin plays a significant role in various biological processes, including differentiation, development, proliferation, and oxidative stress, which are regulated by microRNAs (36). Moreover, scientific studies have demonstrated that it can effectively hinder the creation of inflammatory proteins such as tumor necrosis factor-alpha (TNF-α), interleukin-1 (IL-1), IL-6, IL-8, and IL-12, in addition to obstructing the activity of nuclear factor kappa B (NF-kB) and enzymes responsible for generating harmful reactive molecules like lipoxygenase, cyclooxygenase, and nitric oxide synthases (37, 38). Curcumin plays a significant role in reducing the production of oxygen free radicals by decreasing malondialdehyde (MDA) levels and increasing the expression of genes responsible for the production of enzymes such as glutathione peroxidase (GPX-4 and GPX-1), manganese SOD, copper/zinc-SOD, and CAT (36) (**Figure 2**). Decreases in the functioning of vascular endothelial cells due to aging have been associated with a greater likelihood of developing cardiovascular disorders. Changes in lifestyle, specifically increasing aerobic activity and making dietary adjustments, can have a beneficial effect on the process of vascular aging. Curcumin, a key compound found in turmeric, is widely recognized for its powerful abilities to combat inflammation and act as an antioxidant. The study involved an investigation of flow-mediated dilation, which is a measure of endothelial function, in postmenopausal women, and how this was influenced by both the consumption of curcumin and participation in aerobic exercise training. Thirty-two postmenopausal women were divided into three groups based on their assignment: the control group, the exercise group, and the curcumin group. The curcumin group ingested curcumin orally for a duration of eight weeks, while the exercise group engaged in moderate aerobic exercise training for the same period. The research did not find any noticeable distinctions between the groups when it comes to initial flow-mediated dilation or other crucial outcome measures. Flow-mediated dilatation significantly increased in both the exercise and curcumin groups, while no noticeable changes were observed in the control group (39) (**Table 1**).

**Table 1:** The effects of polyphenols along with exercise in postmenopausal women.

Intervention	Study model & population	Study design	Dosage & protocol	Duration	Main outcomes	Reference
Curcumin	Human; Postmenopausal women (n=32)	RCT	Curcumin: 150 mg/day; Aerobic training (moderate intensity)	8 weeks	Increased flow-mediated dilation significantly in combined group.	(39)
Curcumin	Human; Postmenopausal women (n=204)	RCT	Curcumin: 150 mg/day; Aerobic training (moderate intensity)	8 weeks	Increased central arterial compliance; greatest improvement in combined group.	(40)
Curcumin	Human; Sedentary overweight women (n=44)	RCT	Curcumin: 1.5 mg/kg; Pilates training	12 weeks	Increased SIRT1 levels; significantly improved body composition (weight and fat %).	(41)
Curcumin	Animal; Ovariectomized rats with obesity	Experimental	Curcumin supplement; Moderate intensity interval training	8 weeks	Decreased cardiac risk factors (ICAM-1, Total Cholesterol).	(42)
Curcumin	Human; Overweight postmenopausal women (n=48)	RCT	Curcumin: 2,100 mg/day; Pilates training (45 min, 3x/week)	12 weeks	Increased Klotho levels; Improved HRQL, physical/mental health. No additive effect of supplement over exercise alone.	(43)
Green Tea	Human; Osteopenic postmenopausal women (n=171)	RCT	GTP: 500 mg/day; Tai Chi (60 min, 3x/week)	24 weeks	Improved emotional/mental quality of life (via Tai Chi); GTP had minimal impact on QoL.	(44)
Green Tea	Human; Overweight/Obese women (n=8)	Randomized Crossover	GTE: 400 mg (acute dose); Walking (65% HRR)	Acute (Single bout)	No significant acute effect on fasting glucose, insulin, TNF-α, or adiponectin.	(45)
Green Tea	Human; Osteopenic postmenopausal women (n=171)	RCT	GTP: 500 mg/day; Tai Chi training	6 months	Significantly reduced urinary 8-OHdG (oxidative stress marker).	(46)
Green Tea	Human; Overweight/Obese women (n=20)	RCT	Green tea: 340 mg/day; Aerobic-resistance training (60 min, 3x/week)	6 weeks	Improved body composition (reduced body fat % and BMI); No change in WHR or cholesterol.	(47)
Green Tea	Human; Sedentary women (n=30)	RCT	Green tea powder: 6 g/day; Water aerobics (50-70% HRmax, 3x/week)	8 weeks	Improved aerobic capacity; No significant additive effect on lipid profile compared to exercise alone.	(48)
Green Tea	Human; Sedentary women (n=24)	RCT	GTE: 1,200 mg/day; Aerobic exercise (Fatmax intensity)	2 weeks	Reduced body weight and BMI; Increased carbohydrate oxidation.	(49)
Soy	Human; Postmenopausal women (n=60)	RCT	Soy protein: 25 g/day; Resistance training (60-80% 1RM)	16 weeks	Increased Resting Energy Expenditure (REE) by 17% in combined group.	(50)
Ginger	Human; Obese postmenopausal women (n=48)	RCT	Ginger extract: 1,500 mg/day; Aerobic training (50-70% HRmax)	24 weeks	Significant improvement in liver enzymes (ALT, AST) and lipid profiles.	(51)

Reduced arterial flexibility associated with aging increases the likelihood of developing heart problems. Altering one’s way of life, particularly when it comes to diet and regular aerobic exercise, has positive effects in delaying the process of arterial aging. Curcumin, an active element found in turmeric, possesses properties that have the ability to reduce inflammation.

#### 4.1.1 Clinical evidence

A separate study investigated the impact of curcumin consumption, both on its own and in combination with an aerobic exercise regimen, on the vascular compliance of postmenopausal women. In this study, a total of 204 postmenopausal women were divided into four groups of 51 members each: those who were assigned to both exercise and placebo (Ex + placebo), those who participated in exercise and received curcumin supplementation (Ex + curcumin), as well as those assigned to receive only a placebo (placebo + placebo). During a period of eight weeks, subjects orally ingested either curcumin or a placebo. The moderate aerobic exercise program was provided to the exercise groups for a duration of eight weeks. The results revealed that the placebo control group did not experience any changes in their carotid arterial compliance, whereas significant improvements were observed in the curcumin, Ex + placebo, and Ex + curcumin groups. The group that received both Ex and curcumin demonstrated the greatest improvements in carotid artery flexibility (40). A study was carried out to evaluate the effects of a 12-week regimen of turmeric consumption and Pilates training on the body fat percentage, weight, and SIRT 1 levels of women in the postmenopausal stage. In this study, a group of 44 women who were both overweight and inactive were randomly divided into two groups. One group received a placebo while the other received a daily dose of 1.5 mg/kg of turmeric powder, along with or without Pilates instruction, for a duration of 12 weeks. According to the findings, implementing a 12-week program that combined Pilates and turmeric supplementation significantly increased serum SIRT1 levels by approximately 2.9 times in the groups that underwent training, as opposed to the control group. This intervention also resulted in reduced weight and body fat percentage of approximately 8% and 6%, respectively. In each of the measurements taken, significant differences were found among the groups that received training versus those in the control or turmeric groups. The two groups, one that practiced Pilates and the other that practiced Pilates with turmeric, showed no significant differences between them. Moreover, upon evaluating the dependent variables of the turmeric supplementation group in relation to the control group or initial measurements, no noticeable disparities were observed (41). Different research examined the impacts of consuming turmeric every day and undergoing Pilates exercise guidance for 12 weeks on the serum Klotho levels and overall QoL in women who are in their middle age and overweight. A group of 48 postmenopausal women who were overweight were selected, and they were then divided into four groups through a random process: a control group, a group that performed Pilates exercise, a group that received turmeric supplementation, and a group that received both Pilates exercise and turmeric supplementation. During the twelve-week Pilates program, participants engaged in a 45-minute workout at an intensity level between 40% and 80% of their heart rate reserve. These workout sessions were held three times a week. As stated in the provisions of the supplement agreement, a daily dosage of 2,100 mg of turmeric powder was required. The findings clearly showed that participating in Pilates classes for a period of 12 weeks, with or without the addition of turmeric supplements, significantly increased the levels of Klotho and improved overall QoL, physical and mental well-being, and social functioning. However, when looking at these variables, adding turmeric supplementation did not show any significant changes (43).

#### 4.1.2 Preclinical evidence

In a preclinical study, Hosseini (42) investigated the combined effects of moderate-intensity exercise and curcumin supplementation in ovariectomized Wistar rats, a model mimicking postmenopausal adiposity. Following an 8-week protocol, the combination group exhibited significantly lower levels of Intercellular Adhesion Molecule-1 (ICAM-1) and total cholesterol compared to controls. However, it is important to note that Vascular Cell Adhesion Molecule-1 (VCAM-1) levels were not significantly altered, suggesting that while curcumin may attenuate specific inflammatory pathways, its effect on overall vascular adhesion markers in this animal model is selective. Furthermore, the translation of these rodent-derived vascular benefits to clinical cardiovascular endpoints in women remains to be fully established (42).

### 4.2 The beneficial effects of exercise and green tea intake in postmenopausal women

Studies suggest that engaging in regular sessions of Tai Chi and incorporating green tea polyphenols into one’s diet can potentially contribute to strengthening the bones of women with osteopenia and enhancing their metabolic profiles in menopausal phase (**Figure 2**).

#### 4.2.1 Clinical evidence

Research was conducted to evaluate the wellbeing and effect on overall well-being of women with low bone density after menopause, who incorporated GTP with TC exercise for a period of 24 weeks. In a span of 24 weeks, a group of 171 women who have reached menopause and were identified with low bone density were randomly divided into four different treatment plans: receiving a placebo (500 mg of starch per day), consuming green tea polyphenols (500 mg of GTP per day), taking a placebo along with participating in tai chi training (three 60-minute sessions per week), and taking green tea polyphenols while engaging in tai chi training. To ensure the safety of participants, levels of liver enzymes (alanine and aspartate aminotransferases), alkaline phosphatase, and total bilirubin were regularly tested at the beginning and every 4 weeks throughout the study. The SF-36 survey was utilized in order to evaluate the overall well-being at the start of the study, as well as after 12 and 24 weeks have passed. The findings indicated that throughout the study, neither TC training nor GTP intake had any influence on indicators associated with the functioning of the liver or kidney. None of the participants experienced any negative consequences associated with the study treatment. Participation in TC exercise significantly improved the subjects' emotional and mental well-being, but GTP supplementation had minimal impact on their overall QoL (44). Dostal et al. (45) examined the acute effects of Green Tea Extract (GTE) combined with exercise on glucose tolerance in sedentary postmenopausal women. A significant limitation of this study was its small sample size (n=8) and acute design (single-dose administration). The results indicated that neither GTE nor the combination intervention significantly altered insulin or glucose area-under-the-curve (AUC) responses during an oral glucose tolerance test. The lack of significant findings may be attributable to the limited statistical power or the insufficiency of a single acute dose to modulate established metabolic inflexibility in this demographic (45).

The study examined the impact of GTP and TC on reducing oxidative harm in women who were postmenopausal and had osteopenia. A research conducted for a period of 6 months involved 171 postmenopausal women diagnosed with osteopenia, living in Lubbock County, Texas. They were randomly assigned to receive either a placebo or one of the following treatments: 500 mg of GTP daily, a combination of 500 mg of GTP daily and 60 minutes of group exercise three times a week, or a combination of 500 mg of GTP daily and group exercise three times a week. The findings demonstrated that the three treated groups experienced significantly reduced levels of urine 8-OHdG during both the 3-month and 6-month interventions, when compared to the placebo group. The groups that received interventions of GTP, TC, and GTP+TC demonstrated significant effects in reducing the biomarker of oxidative damage, considering both the duration of intervention and the dosage used (46). A separate study analyzed the impact of consuming green tea and engaging in both aerobic and resistance exercise on the lipid levels and physical composition of overweight and obese women who had gone through menopause. The group of individuals chosen for the study were twenty women who were menopausal volunteers. Through a process of random selection, they were divided into two groups – the experimental group, which consisted of individuals who drank green tea and participated in aerobic resistance exercise, and the control group. Over the course of six weeks, the participants in the intervention group engaged in aerobic-resistance training for three 60-minute sessions each week and consumed 340 mg of green tea every day. Prior to the completion of the intervention period, the participants underwent examinations to assess any alterations in their serum lipid levels and body composition. Following the conclusion of the intervention, the same tests were conducted to determine if any changes occurred. After participating in a 6-week program, the results of the analysis on body composition revealed a reduction in both the percentage of body fat and BMI. There were no significant changes observed in WHR and blood cholesterol levels in the intervention group (47).

In separate research, the effects of participating in water-based aerobic activity, with and without the addition of green tea, were analyzed in relation to various factors that contribute to heart disease risk, and the physical makeup of women who have gone through menopause and are generally sedentary. A group of thirty women who had reached menopause and were not regularly physically active were blindly assigned to one of three groups: water-based aerobic exercises, consumption of green tea, and a combination of exercise and green tea. The aerobic exercise regimen for both the exercise group and exercise group plus green tea included three sessions per week for a duration of eight weeks, performed in water, with an intensity level ranging from fifty to seventy percent of the maximum heart rate. The study involved participants who were assigned to both exercise groups and consumed green tea three times per day, resulting in a total intake of six grams of dry green tea powder. According to the results of the research, it was proven that green tea did not impact the liposomal and tonicity measures in any way. Each of the three groups experienced an advancement in their aerobic capacity, although there were no noticeable variations between the groups (48). In a separate investigation, a total of 24 women who led inactive lifestyles and had reached menopause were divided into two distinct categories: one group received a combination of exercise and supplements while the other received a combination of exercise and a fake treatment. The participants in the supplement group consumed 1200 mg of green tea extract daily in the form of capsules. The training program consisted of four weekly sessions of aerobic exercise for two weeks, with each session lasting 40 to 50 minutes and conducted at a level where fat burning was at its maximum. After a span of two weeks, both groups experienced a significant decrease in weight, BMI, waist to hip ratio, average total carbohydrate oxidation, and HDL-cholesterol levels. Despite an increase in these measures, including average total fat oxidation, Fatmax, maximal fat oxidation, and peak oxygen consumption, their overall values remained fairly consistent and didn’t experience significant change. The triglyceride and visceral fat levels showed a considerable decrease solely in the group that received exercise and placebo treatment, while the LDL-cholesterol levels saw a significant increase only in the exercise and supplement group. In both of the groups, there was also no statistically significant decrease in body fat percentage (49).

### 4.3 The beneficial effects of exercise and soy intake in postmenopausal women

A previous report has indicated that soy can bring about advantageous outcomes in postmenopausal women. The main objective of the research was to examine how the combination of soy protein intake and weight-training exercises impacts the resting energy expenditure (REE) of women in the postmenopausal stage.

#### 4.3.1 Clinical evidence

A total of sixty female individuals were involved in a sixteen-week clinical trial, wherein they were divided into four distinctive groups: G1, consisting of those who received soy protein and participated in physical exercise; G2, comprising individuals who were given a placebo and engaged in exercise; G3, made up of participants who received soy protein but did not partake in any physical activity; and G4, composed of individuals who were given a placebo and did not engage in exercise. Randomly, participants were given either 25 g/day of soy protein or a placebo consisting of maltodextrin. In three weekly sessions, ten weight-based exercises were undertaken, each set consisting of 8–12 repetitions, totaling 60-80% of one’s maximum effort. The REE was calculated using the O2 and CO2 levels, which were measured via indirect calorimetry for a duration of 30 minutes at controlled humidity and temperature. Significantly, there was a noticeable rise in the REE in G1 and G2, reaching 17% and 9% respectively, while G4 saw a decrease of 4% (50). Choquette et al. (52) conducted a 6-month trial involving 70 postmenopausal women. While exercise independently improved muscle strength, the addition of soy isoflavones (70 mg/day) did not yield significant additive benefits for muscle function or bone density. This null finding highlights a potential threshold effect; the dosage used may have been insufficient to elicit an osteogenic response in this specific cohort, or the intervention duration was too short to capture slow-turning bone metabolic changes (52). Llaneza et al., investigated the role of soy isoflavones on insulin resistance of post-menopausal women. In this study one group received 40 mg of soy isoflavones along with regular exercise and Mediterranean diet (intervention group) while the other group only received the latter two interventions (control group). Results revealed that compared to control group, soy isoflavones improved Mean homeostasis model assessment of insulin resistance of subjects (53).

### 4.4 The beneficial effects of exercise and ginger intake in postmenopausal women

Ginger (*Zingiber officinale*) has been utilized historically for its antiemetic and anti-inflammatory properties (54). Its rhizome contains potent bioactive compounds, including gingerols, shogaols, and zingerone, which modulate lipid metabolism and insulin sensitivity (55, 56). Unlike the extensive literature on soy or calcium, evidence regarding ginger’s specific efficacy for postmenopausal symptoms is emerging and less robust. However, mechanistic studies suggest it may enhance hepatic cholesterol 7-alpha-hydroxylase activity, thereby facilitating the conversion of cholesterol into bile acids (57, 58).

However, the findings of Talaee and colleagues revealed that despite no notable contrast between the two groups, there was a decrease in the experimental group’s LDL and LDL to HDL cholesterol ratio after consuming 3 grams of zingiber powder capsules per day for 8 weeks (59). The zingiber plant effectively reduces lipid levels and facilitates the transformation of cholesterol into bile acids by increasing the activity of Hepatic cholesterol 7 hydrolase enzymes, ultimately restricting the production of bile acids (16). Menopause is a natural phase that women experience in their lives, and engaging in consistent physical activity aids in averting issues and illnesses such as liver disease. By considering the potential benefits of zingiber and regular physical training in reducing obesity, while also factoring in the probable outcome of their combined impact. The main goal of the research was to evaluate the impact of aerobic exercise and ginger extract on the lipid profiles, body composition, and specific liver enzymes of women who were both overweight and in menopause. Four sets of 48 women in their menopausal stage who were obese were given random assignments to four different groups: control, aerobic training, aerobic training with ginger extract, and aerobic training with control. Over the course of 24 weeks, the participants engaged in aerobic exercise at a maximum heart rate of 50-70% for 60 minutes, three times a week. Additionally, they consumed 500 mg of ginger extract three times daily. The results clearly showed a significant decrease in ALT and AST levels among obese menopausal women who underwent 12 and 24 weeks of ginger and ginger-training exercises. Furthermore, women who were overweight and going through menopause and participated in ginger training for 12 and 24 weeks experienced positive changes in their cholesterol levels and physical composition (51).

## 5 Synergies targeting musculoskeletal and cardiometabolic health

### 5.1 The beneficial effects of vitamin D intake combined with exercise in postmenopausal women

**Figure 3:**
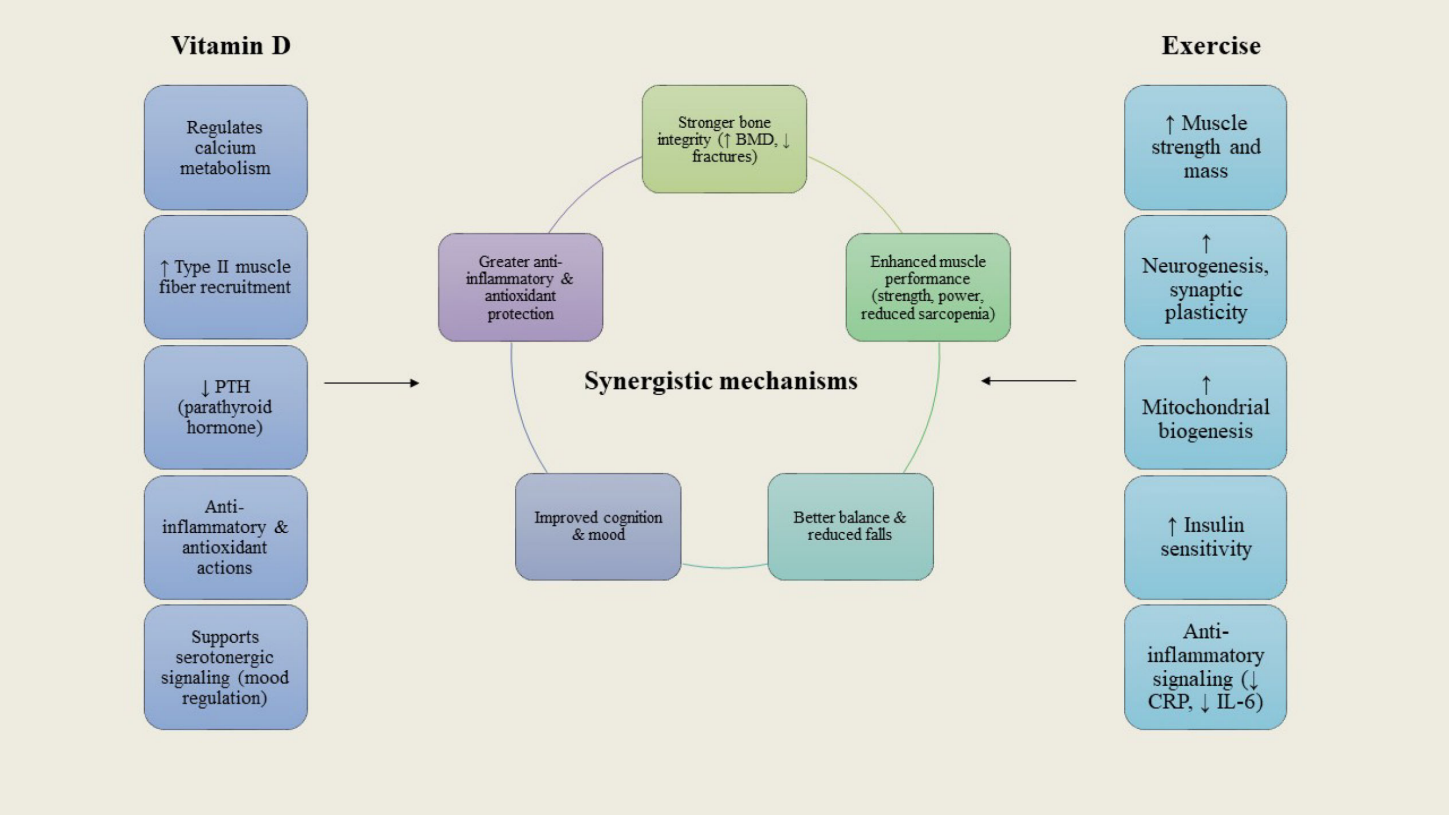
Interactions between vitamin D and exercise in postmenopausal women. While vitamin D regulates calcium metabolism, muscle fiber recruitment, and mood pathways, exercise improves strength, neurogenesis, and metabolic control. Their overlap yields amplified benefits for bone integrity, muscle function, cognition, and anti-inflammatory protection.

Aging in postmenopausal women is frequently associated with a multifaceted array of physiological and psychological transformations that have detrimental implications for physical functioning, metabolic homeostasis, and mental well-being. These transformations encompass sarcopenia, osteoporosis, insulin resistance, dyslipidemia, affective disorders, and an overarching deterioration in QoL. Among the numerous strategies proposed to mitigate age-related decline, integrative methodologies that amalgamate lifestyle modifications, such as exercise regimens, with nutritional supplementation have attracted increasing scholarly attention. Significantly, the synergy of consistent physical activity and vitamin D supplementation emerges as a viable, non-pharmacological intervention to facilitate healthy aging in postmenopausal women. Prevailing evidence indicates that the combined effects of exercise and vitamin D may enhance the advantages of either intervention implemented in isolation. For instance, vitamin D has the potential to augment the effectiveness of physical exercise on muscular functionality by influencing calcium metabolism and enhancing the recruitment of type II muscle fibers, a factor that holds significant importance in the prevention of sarcopenia and falls. Furthermore, both interventions demonstrate anti-inflammatory and antioxidant properties, mitigate systemic oxidative stress, and alter signaling pathways that are integral to mitochondrial biogenesis, insulin sensitivity, and neuroplasticity. From a psychological perspective, this comprehensive approach may also alleviate the prevalence of depressive symptoms and anxiety, conditions that frequently remain underdiagnosed among postmenopausal women. Physical exercise stimulates the release of endorphins and promotes neurogenesis, whereas vitamin D influences serotonergic neurotransmission and provides neurotrophic support, thereby contributing to enhanced mood and cognitive resilience (**Figure 3**).

#### 5.1.1 Clinical evidence

A randomized controlled trial was conducted to assess the synergistic effects of water-based aerobic exercise and vitamin D3 supplementation on BMI and physical performance metrics in overweight or obese postmenopausal women exhibiting deficient vitamin D levels. Participants were systematically allocated into four distinct groups: exercise in conjunction with vitamin D, exercise exclusively, vitamin D solely, and a control group. Following an 8-week intervention period, the cohort receiving both exercise and vitamin D demonstrated the most pronounced enhancements in BMI, handgrip strength, gait velocity, and postural stability. The exercise-only group similarly exhibited improvements in strength, balance, and gait velocity, whereas the vitamin D-only group demonstrated an increase in handgrip strength when compared to the control group (60) (**Table 2**). Another investigation was conducted to examine the impact of aquatic training and vitamin D3 supplementation on femoral BMD, serum 25-hydroxyvitamin D (25(OH)D), and parathyroid hormone (PTH) levels in postmenopausal obese women exhibiting vitamin D insufficiency. The subjects were systematically allocated into four distinct groups: aquatic training with vitamin D3 supplementation (ATD), aquatic training alone (AT), vitamin D3 supplementation alone (D), and a control group (CON). Following an 8-week intervention period, the ATD cohort demonstrated the most pronounced enhancement in femoral BMD and serum 25(OH)D concentrations, in addition to the most considerable decrease in PTH levels. The group engaging in aquatic training exclusively also exhibited improvements in BMD and reductions in PTH, whereas the vitamin D3 supplementation group alone resulted in elevated levels of 25(OH)D and a decrease in PTH when compared with the CON (61).

**Table 2:** The effects of vitamin D and omega-3 along with exercise in postmenopausal women.

Nutrient	Model	Dosage	Duration	Main effects	Molecular/Functional targets	Ref
vitamin D	*In vivo* (Human, postmenopausal women; n = 40)	Vitamin D3 supplementation (dosage not specified in provided text); Water-based aerobic exercise (3x/week)	8 weeks	- ↓ BMI - ↑ Handgrip strength - ↑ Gait speed - ↑ Static balance (both legs) in WTD group - Moderate improvements in WT and D groups	- Vitamin D3 → Modulates muscle strength and neuromuscular function - Exercise → Improves balance, mobility, and muscle performance	(60)
vitamin D	*In vivo* (Human, postmenopausal obese women; n = 40)	- Vitamin D3 supplementation: 4000 IU/day (oral) - Aquatic training: aerobic exercises 3x/week	8 weeks	- ↑ Femur BMD in ATD > AT > D > CON - ↑ Serum 25(OH)D in ATD > D > AT > CON - ↓ PTH in ATD < AT < D < CON	- Vitamin D3: ↑ Serum 25(OH)D → ↓ PTH secretion; supports calcium homeostasis - Exercise: Stimulates bone formation and improves mechanical loading on bone tissue	(61)
vitamin D	*In vivo* (Human, healthy postmenopausal women; n = 44)	- Vitamin D3: 50,000 IU every 2 weeks (oral) - Resistance Training (RT): 3–4 sets, 70–85% 1RM, 3×/week (leg press, chest press, leg extension, leg curl, shoulder press)	12 weeks	- ↑ Upper and lower body muscle strength in RT + VitD and RT + PLA - No significant changes in CAF and NT-3 in any group - VitD alone did not enhance muscle strength or power	- RT: Induced neuromuscular adaptations and improved muscle function - Vitamin D: No additive effect on muscle strength; no impact on CAF or NT-3 levels - CAF and NT-3: Not responsive biomarkers for neuromuscular changes in this context	(62)
vitamin D	*In vivo* (Human, postmenopausal women with osteopenia/osteoporosis; n = 39)	- G1: High-impact training only - G2: High-impact training + calcium + vitamin D - G3: Brisk walking + calcium + vitamin D (Dosage of supplements not specified)	2 years	- G2: ↑ Femoral neck and lumbar spine BMD (T-score BC > 20%), ↓ fracture incidence - G1 & G3: Maintained BMD - Similar fall rates across groups	- Likely targets include pathways related to bone remodeling, such as RANK/RANKL/OPG axis, Wnt/β-catenin signaling, and vitamin D–regulated calcium metabolism (not directly measured in this study)	(63)
vitamin D	*In vivo* (Human; postmenopausal women with metabolic syndrome; n = 46)	- Vitamin D: 50,000 IU (form not specified, likely cholecalciferol), weekly - Aerobic Training: 40–60 min, 60–75% HRmax, 3x/week	8 weeks	- AT + Vit D: ↓ CRP, ↓ IL-6, improved metabolic syndrome indices (e.g., waist circumference, lipid profile, fasting glucose) - AT or Vit D alone: Moderate improvements	- CRP, IL-6 (key pro-inflammatory cytokines) - Likely involvement of NF-κB pathway, vitamin D receptor (VDR) signaling, and AMPK-related metabolic pathways	(64)
vitamin D	*In vivo* (Human; postmenopausal women with T2DM; n = 35)	Vitamin D: 1000 IU/day (oral)	12 months	- ↑ Serum vitamin D levels - ↑ Muscle strength - ↑ Muscle function - Prevention of fragility associated with T2DM and aging	- Vitamin D Receptor (VDR) signaling - Potential modulation of muscle protein synthesis pathways (e.g., via mTOR) - Indirect effects on glucose metabolism and insulin sensitivity	(65)
vitamin D	*In vivo* (Human; postmenopausal women aged 50–70 years with vitamin D deficiency)	Vitamin D Replacement: Dosage not specified, targeted serum level >75 nmol/L Exercise: Core and balance training	8 weeks	- ↑ Balance (via Berg Balance Test and Biodex system) in all intervention groups (except mediolateral stability in Group I) - ↑ Quality of Life (QoL) across multiple NHP domains - No additive/synergistic effect of combining vitamin D and exercise observed	- Vitamin D Receptor (VDR) activation - Potential modulation of neuromuscular coordination and postural control pathways - Indirect influence on central nervous system plasticity and musculoskeletal health	(66)
vitamin D	*In vivo* (Ovariectomized female Wistar rats; n = 72)	Calcium: 35 mg/kg/day Vitamin D: 10,000 IU Exercise: Regular resistance training	Post-intervention duration not specified explicitly; interventions began 2 months after ovariectomy	- ↑ Muscle cell number (especially in Ex + Vit. D + Ca, Vit. D + Ex, Vit. D groups) - ↑ Inflammatory cell number in several intervention groups - ↑ Muscle fiber diameter (notably in Ex + Vit. D + Ca and Vit. D + Ex) - ↓ Endomysium thickness in Ex + Vit. D + Ca and Vit. D + Ex - ↑ Degenerative collagen fiber area in Ex + Vit. D + Ca and Vit. D + Ex	- Vitamin D Receptor (VDR) activation - Calcium-regulated signaling pathways affecting muscle structure - Potential modulation of inflammatory markers, myogenic regulatory factors (MRFs) like MyoD and Myogenin - ECM remodeling via collagen regulation and muscle regeneration pathways	(67)
Vitamin D	*In vivo* (Ovariectomized female Sprague-Dawley rats; n = 72)	Vitamin D: 10,000 IU Calcium: 35 mg/kg/day Exercise: Regular resistance training	Post-ovariectomy duration (exact timeframe not clearly stated; presumed several weeks)	- ↑ Bone mineral density (tail, hip, lumbar), especially in Ex + Vit D group - ↑ Bone thickness in Ex + Vit D group - ↓ Osteoclast numbers in Ca + Vit D, Ex + Ca, Ex + Vit D, Ex + Ca + Vit D groups - ↑ Osteocyte numbers in Ex + Vit D group - No significant changes in bone mineral content of hip/lumbar	- Potential activation of Vitamin D Receptor (VDR) in bone - Regulation of RANK/RANKL/OPG pathway (osteoclastogenesis) - Calcium homeostasis signaling via PTH/VDR - Mechanical loading-induced Wnt/β-catenin signaling promoting osteogenesis - Modulation of osteoblast differentiation markers such as Runx2 and Osterix	(68)
Omega-3	*In vivo* (Aged female mice, 18 months old) Ex vivo (PBMCs from postmenopausal women with overweight/obesity)	DHA-rich n-3 PUFA supplementation (exact dose not specified) Exercise: Long-term treadmill training in mice; Progressive resistance training in women	16 weeks	- Mice: ↑ Hepatic SIRT1 expression (especially with exercise + DHA); ↓ Lipogenic and pro-inflammatory gene expression; ↑ Fatty acid oxidation genes - FOXO1 expression correlated with ↑ hepatic triglycerides and inflammation - Humans: DHA ↑ FOXO1 in PBMCs; RT ↑ SIRT1 in PBMCs; ↓ MASLD biomarkers and fat mass in all intervention groups	- SIRT1: Promotes fatty acid oxidation and metabolic regulation - FOXO1: Regulates lipid metabolism, correlated with hepatic steatosis - ↓ Expression of lipogenic genes (e.g., SREBP-1c, FAS) - ↓ Pro-inflammatory markers (e.g., TNF-α) - ↑ Lipolytic gene (Hsl) expression - Modulation of metabolic and inflammatory pathways in liver and blood	(69)
Omega-3	*In vivo* (human)	DHA-rich n-3 PUFA supplement (dose not specified), supervised resistance training (RT) program	16 weeks	- Moderate reduction in body weight and fat mass in all groups - RT groups maintained bone mineral content, increased upper limb lean mass, decreased lower limb fat mass, increased muscle strength and quality - RT improved glucose tolerance (lower OGTT incremental AUC) - DHA-rich supplement lowered diastolic blood pressure, circulating triglycerides, and increased lower limb muscle quality - No synergistic effect of combined RT and DHA supplementation	- Improved muscle quality and strength - Improved glucose metabolism (OGTT) - Cardiovascular health markers: diastolic blood pressure, triglycerides	(70)
Omega-3	*In vivo* (human)	Omega-3 supplementation: 1000 mg/day	16 weeks	- Increased serum calcitonin (CT) in E+S, E, and S groups - Decreased parathyroid hormone (PTH) in E+S and E groups - Increased estrogen levels in E+S and E groups - No significant change in blood ionized calcium (Ca^2+^) levels	- Regulation of calcium metabolism via increased CT and decreased PTH - Modulation of estrogen levels impacting bone metabolism	(71)
Omega-3	*In vivo* (human)	Fish oil (FO) supplementation (dose not specified) + resistance exercise training (RET)	8 weeks	- Improved physical function in both RET group - Increased handgrip strength only in RET+FO group - RET+FO significantly reduced blood pressure (BP), triglycerides (TG), inflammatory cytokines (TNF-α, IL-6), and oxidative stress markers (MDA, 8-OHdG) - No significant changes in RET-placebo group	- Inflammation: ↓ TNF-α, ↓ IL-6 - Oxidative stress: ↓ MDA, ↓ 8-OHdG - Cardiometabolic: ↓ BP, ↓ TG - Muscle function: ↑ handgrip strength	(72)

A research investigation examined the ramifications of a 12-week regimen of resistance training (RT) combined with vitamin D supplementation on muscle strength and specific biomarkers (CAF and NT-3) in postmenopausal females. A total of forty-four subjects were systematically classified into four distinct cohorts: RT + placebo, vitamin D alone, RT + vitamin D, and placebo. Although the administration of vitamin D in isolation did not yield improvements in muscle strength, both RT cohorts (regardless of vitamin D supplementation) exhibited marked enhancements in both upper and lower body strength and power. Nevertheless, no noteworthy alterations were detected in CAF or NT-3 concentrations across any of the experimental groups. The incorporation of vitamin D did not further augment the strength improvements beyond those realized through resistance training in isolation (62). A comprehensive investigation assessed the impact of three distinct interventions on BMD in postmenopausal females diagnosed with osteopenia or osteoporosis. The study participants were systematically allocated to either high-impact training (Group 1), high-impact training supplemented with calcium and vitamin D (Group 2), or vigorous walking combined with calcium and vitamin D (Group 3). Throughout a span of two years, all groups either preserved or enhanced their BMD; however, Group 2 exhibited the most significant enhancement in T-scores for both the femoral neck and lumbar spine (exceeding 20%). The incidence of fractures was found to be the lowest in Group 2, whereas the rates of falls were comparable across all groups. This finding indicates that the synergistic effect of high-impact exercise coupled with calcium and vitamin D supplementation is the most efficacious approach for augmenting BMD and mitigating fracture risk (63).

A research study assessed the anti-inflammatory ramifications of aerobic exercise in conjunction with vitamin D supplementation among postmenopausal women diagnosed with metabolic syndrome. A total of forty-six subjects were systematically categorized into four distinct groups: aerobic training combined with vitamin D (50,000 IU), aerobic training exclusively, vitamin D supplementation alone, and a control group. Throughout an 8-week duration, the group receiving the combination intervention exhibited the most pronounced reductions in inflammatory biomarkers (CRP and IL-6) alongside the most substantial enhancements in indices associated with metabolic syndrome. Although both exercise and vitamin D administered independently yielded positive outcomes, their synergistic application engendered the most potent anti-inflammatory and metabolic benefits (64). A longitudinal investigation spanning twelve months assessed the ramifications of daily vitamin D supplementation (1000 IU) on physical fitness parameters among postmenopausal women diagnosed with type 2 diabetes mellitus. A cohort of thirty-five participants exhibited elevated serum vitamin D concentrations, enhanced muscular strength and functionality, as well as improved management of frailty correlated with the aging process and diabetes. The results indicated that vitamin D supplementation in isolation can exert a favorable influence on physical fitness and assist in the prevention of functional deterioration within this specific demographic (65).

A research investigation was conducted to assess the impact of vitamin D supplementation and core/balance exercises on balance, fall risk, and QoL among postmenopausal women exhibiting deficient levels of vitamin D. The participants were categorized into three distinct intervention groups: one receiving vitamin D supplementation exclusively, another engaging in exercise solely, and a third group partaking in a combination of both interventions, with a fourth cohort (characterized by adequate vitamin D levels) functioning as the control group and participating in exercise. Following an 8-week intervention period, all groups manifested enhancements in balance and QoL, with the exception of a lack of significant improvement in mediolateral stability within the vitamin D-only group and in QoL for the exercise-only cohort. No noteworthy differences were detected among the three intervention groups following the treatment; however, the control group exhibited superior baseline scores (66). Another investigation examined the impact of resistance training in conjunction with vitamin D and calcium supplementation on muscle tissue in ovariectomized rats, which serve as an experimental model for postmenopausal conditions. Seventy-two female rats were systematically allocated to various intervention groups. The findings indicated that the cohorts subjected to both exercise and vitamin D (with or without calcium) exhibited an increase in muscle cell quantity and greater muscle fiber diameters in comparison to the control group. Furthermore, inflammatory cell counts were observed to elevate in the majority of the intervention groups. Moreover, the synergistic effect of exercise combined with vitamin D and calcium resulted in a reduction of endomysium thickness and an expansion of degenerative collagen fiber areas, thereby implying a structural remodeling of the muscle tissue. The integration of exercise and vitamin D yielded the most pronounced beneficial effects on muscle morphology (67).

#### 5.1.2 Preclinical evidence

A research investigation assessed the impact of resistance training in conjunction with vitamin D and calcium supplementation on skeletal health in ovariectomized postmenopausal rats. A total of seventy-two rats were allocated into nine distinct groups, each subjected to varying interventions. The findings revealed a significant enhancement in BMD within the tail, hip, and lumbar regions in the exercise combined with vitamin D cohort compared to the control subjects, whereas bone mineral content exhibited no significant alterations. Furthermore, the thickness of bone was observed to be most pronounced in this specific group. In addition, the number of osteoclasts (cells responsible for bone resorption) diminished in several of the supplemented and exercise groups, while the population of osteocytes (cells that maintain bone integrity) increased solely in the exercise plus vitamin D group, thereby suggesting an improvement in bone quality with this particular combination (68).

Collectively, these combined benefits contribute to a higher QoL and reduced risk of age-related diseases. As part of a broader personalized lifestyle intervention, this integrative approach holds significant promise for optimizing long-term health outcomes in aging female populations.

### 5.2 The beneficial effects of omega-3 intake combined with exercise in postmenopausal women

**Figure 4:**
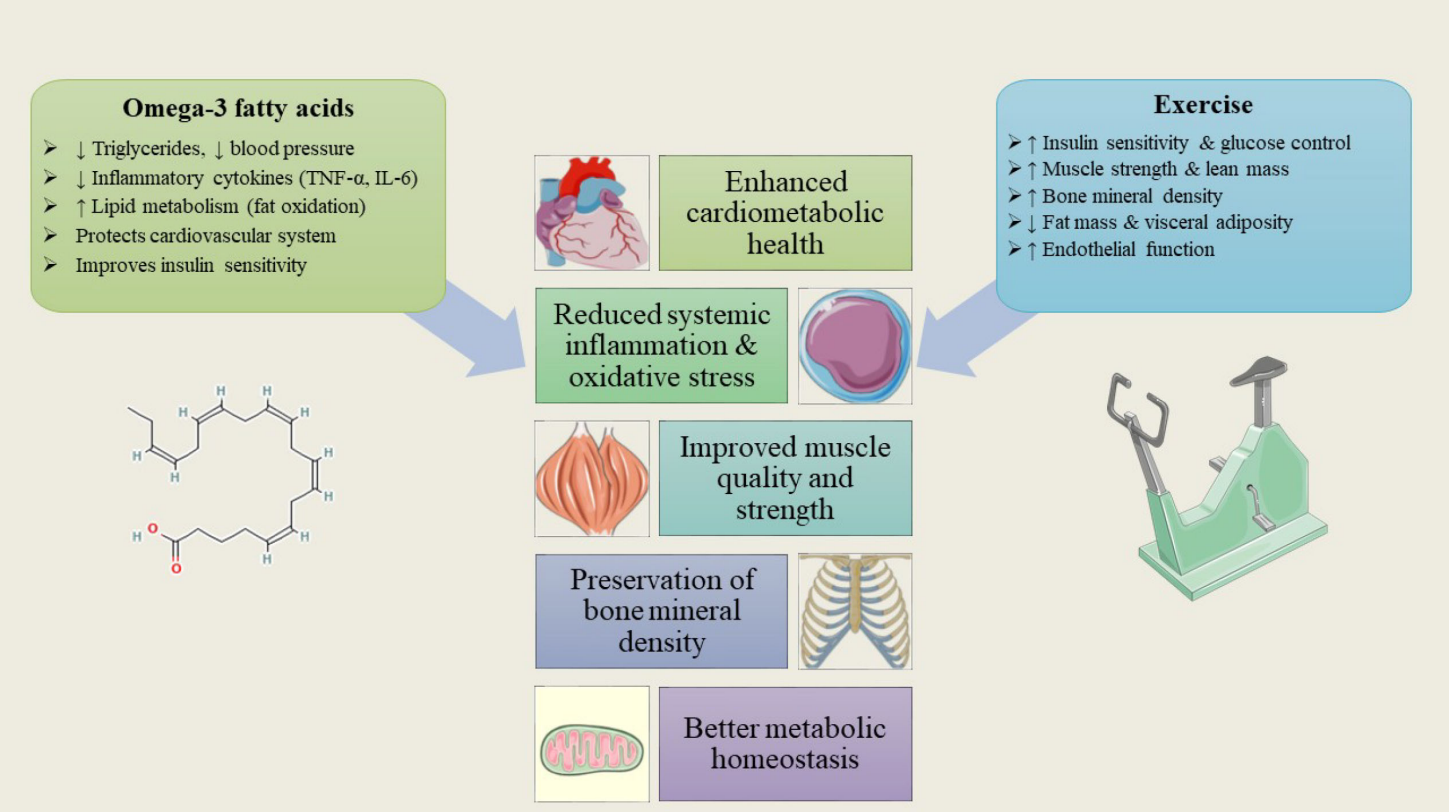
Complementary effects of omega-3 fatty acids and exercise. Omega-3s lower inflammation, triglycerides, and blood pressure, while exercise boosts insulin sensitivity, lean mass, and bone strength. Together, they enhance cardiometabolic health, preserve muscle and bone, and reduce chronic disease risk in postmenopausal women.

Postmenopausal females encounter heightened susceptibilities to metabolic, cardiovascular, and musculoskeletal pathologies attributable to hormonal fluctuations and the aging process. The integration of omega-3 fatty acid consumption with habitual physical activity represents a potentially effective approach to alleviate these vulnerabilities. Omega-3 fatty acids are recognized for their anti-inflammatory and cardioprotective characteristics, which contribute to the enhancement of muscular function, the optimization of lipid profiles, and the reduction of oxidative stress levels. When combined with physical exercise, particularly resistance or aerobic training modalities, omega-3 supplementation significantly magnifies the advancements in muscle strength, bone integrity, metabolic homeostasis, and cardiovascular performance. This synergistic effect fosters healthier aging, diminishes the prevalence of chronic illnesses, and enhances the overall QoL for postmenopausal women (**Figure 4**).

The investigation examined the contributions of SIRT1 and FOXO1 gene expression in the context of obesity-related metabolic dysfunction-associated steatotic liver disease (MASLD) utilizing aged obese murine models and postmenopausal females experiencing overweight/obesity. In the murine subjects, elevated levels of Sirt1 were found to be positively correlated with enhanced fatty acid oxidation and diminished fat synthesis and inflammatory responses, while both physical exercise and DHA supplementation were shown to augment Sirt1 expression. In contrast, the expression of Foxo1 was associated with increased hepatic lipid accumulation and inflammatory processes.

#### 5.2.1 Clinical evidence

In human leukocytes, the expression of FOXO1 exhibited an inverse correlation with indices of hepatic fat. Following a 16-week regimen of DHA supplementation and/or resistance training, biomarkers indicative of MASLD and overall body fat exhibited reductions across all groups; however, the differences between the various interventions did not reach statistical significance. Importantly, DHA was observed to elevate FOXO1 expression, whereas resistance training was associated with an increment in SIRT1 levels within human cells, underscoring their prospective roles in the enhancement of metabolic health (69). Another investigation concerning overweight and obese postmenopausal women assessed the impact of resistance training (RT) and supplementation with DHA-enriched n-3 polyunsaturated fatty acids over a duration of 16 weeks. While all experimental groups exhibited moderate reductions in body weight and adipose tissue, individuals engaged in the RT regimen demonstrated preservation of BMD, an increase in upper limb lean mass, a decrease in lower limb adiposity, as well as enhancements in muscle strength and quality in comparison to their non-exercising counterparts. Furthermore, RT significantly improved glucose tolerance. The administration of DHA supplementation resulted in reductions in diastolic blood pressure and triglyceride levels, alongside improvements in lower limb muscle quality. Collectively, the synergistic effects of RT and DHA supplementation provide complementary advantages for skeletal muscle function and metabolic health within this demographic (70).

Tartibian et al. (71) reported that omega-3 supplementation combined with aerobic exercise significantly reduced Parathyroid Hormone (PTH) levels. However, a critical limitation of this study was the lack of direct Bone Mineral Density (BMD) measurements; the study relied solely on serum biomarkers, which—while indicative of turnover—do not always correlate perfectly with structural bone preservation (71). A study involving 20 cognitively intact older females assessed the impact of an 8-week regimen of resistance exercise training (RET) supplemented with fish oil (FO) against a control group receiving RET alongside a placebo, focusing on physical functionality and cardiometabolic risk determinants. Both experimental cohorts demonstrated enhancements in physical functionality; however, only the RET-FO cohort exhibited statistically significant improvements in handgrip strength, reductions in both blood pressure and triglyceride levels, as well as decreases in inflammatory biomarkers (TNF-α, IL-6) and oxidative stress indicators (MDA, 8-OHdG). This finding implies that the synergistic effect of FO supplementation in conjunction with resistance training may confer superior benefits to cardiometabolic health in older women compared with resistance exercise alone (72). Overall, the conjunction of omega-3 supplementation with consistent physical exercise yields considerable health advantages for postmenopausal women. This synergistic methodology promotes enhancements in muscular strength, BMD, metabolic functioning, and cardiovascular health, concurrently mitigating inflammation and oxidative stress. Collectively, the consumption of omega-3 fatty acids and the practice of physical exercise present a robust, non-pharmacological intervention aimed at augmenting overall well-being and diminishing the risk of age-related chronic diseases within this susceptible demographic.

## 6 Discussion: the beneficial effects of functional foods and exercise on postmenopausal women

Exercise is beneficial for women of all BMI statuses, but the effects can vary. In normal-weight women, regular physical activity can help maintain healthy metabolic profiles and prevent the onset of menopausal symptoms and chronic diseases such as cardiovascular disease (7, 73–75). For overweight or obese postmenopausal women, exercise not only helps in reducing body weight and fat mass but also improves insulin sensitivity and reduces the risk of developing type 2 diabetes and cardiovascular diseases (76–78). In postmenopausal women, functional foods can help mitigate oxidative stress and inflammation, which are linked to chronic conditions like cardiovascular disease and diabetes (79, 80). The effectiveness of functional foods might be more pronounced in overweight and obese individuals due to higher levels of oxidative stress and inflammation in these groups (81–83). Combining exercise with functional foods intake could have synergistic effects. Exercise increases the body’s natural antioxidant defenses and improves endothelial function, while functional foods can enhance these effects by providing additional antioxidant and anti-inflammatory support (84, 85). This combination may be particularly effective in reducing inflammation and improving metabolic health in postmenopausal women across all BMI categories (84, 86, 87). Exercise and functional foods can affect molecular signaling pathways involved in inflammation, oxidative stress, and metabolism. For instance, both can decrease the activation of NF-kB, a key regulator of inflammation (88–90). While this review focused on polyphenols and phytoestrogens, dietary nitrates (abundant in beetroot and leafy greens) represent a critical emerging area for postmenopausal health. Nitrates enhance nitric oxide (NO) bioavailability, which is often compromised due to estrogen withdrawal, leading to endothelial dysfunction (91, 92). Preliminary evidence suggests that nitrate supplementation, when combined with exercise, may synergistically improve vascular conductance and muscle contractile efficiency by reducing the oxygen cost of exercise (93). Although specific RCTs in postmenopausal cohorts are currently limited compared to athletic populations, the mechanistic rationale for nitrate-exercise synergy in managing age-related vascular stiffness and sarcopenia is strong and warrants prioritized investigation.

The metabolic and molecular responses to exercise and functional foods can differ based on an individual’s BMI. In obese individuals, these interventions might result in significant improvements in insulin sensitivity and reductions in systemic inflammation compared to normal-weight individuals, who may not see as dramatic changes due to having fewer metabolic derangements initially (84, 87). Postmenopausal women typically experience increased oxidative stress and inflammation, contributing to the development of diseases like osteoporosis and atherosclerosis (94, 95). Interventions like exercise and functional foods intake can reduce these harmful processes. The effects can be more significant in overweight and obese women due to higher baseline levels of inflammation and oxidative stress.

## 7 Limitations

Despite the promising evidence presented, several limitations within the current body of literature must be acknowledged.

- Heterogeneity of protocols: There is no standardization regarding the dosage, formulation (e.g., bioavailability of curcumin or green tea extracts), or duration of functional food interventions, making cross-study comparisons difficult. Similarly, exercise protocols vary widely in intensity and modality (aerobic vs. resistance).
- Evidence gap: A significant portion of the mechanistic data is derived from preclinical models (ovariectomized rodents). While valuable for understanding tissue-level changes, these findings do not always translate directly to clinical outcomes in women due to species-specific metabolic differences.
- Biomarker inconsistency: Many clinical trials rely on surrogate biomarkers (e.g., serum osteocalcin or inflammatory cytokines) rather than hard clinical endpoints (e.g., fracture incidence or cardiovascular events), limiting the strength of the conclusions regarding long-term disease prevention.
- Methodological quality: Several included trials suffered from small sample sizes (n < 30) and short durations (< 12 weeks), which may be insufficient to detect changes in slow-turning tissues like bone.
- Scope of review: As a narrative review, this paper provides a qualitative synthesis rather than a quantitative meta-analysis. Consequently, the interpretation of “synergy” is based on reported statistical interactions in individual studies rather than pooled effect sizes.
- Environmental confounders: This review primarily focused on the beneficial effects of bioactive nutrients. However, we did not account for the confounding role of endocrine-disrupting chemicals (EDCs) and food pollutants (e.g., pesticides, plastics) often present in the food supply. Recent evidence suggests that these environmental estrogens can disrupt metabolic homeostasis and contribute to weight gain, potentially masking the beneficial effects of functional foods (96)

## 8 Conclusions

Postmenopausal women face a confluence of physiological and metabolic challenges that impact overall health. This review highlights the potential of integrative lifestyle modifications specifically the combination of structured exercise and functional foods, to address these issues. Current evidence suggests that exercise training improves musculoskeletal robustness and metabolic regulation, while bioactive food components (phytoestrogens, polyphenols, omega-3s) may offer complementary support for inflammation and hormonal balance. While preclinical data demonstrates strong synergistic mechanisms, clinical results vary depending on dosage, intervention duration, and baseline health status. Therefore, while this integrative approach aligns with the principles of preventive medicine, standardized guidelines cannot yet be fully established. Future initiatives should prioritize rigorous, long-term clinical trials to determine the optimal combinatorial protocols necessary to promote healthy aging in this demographic.

## 9 Future perspectives

To advance the field of integrative postmenopausal health, future research must transition from observational associations to rigorous clinical validation. We propose the following priority areas:

- Standardization of protocols: Researchers must establish standardized dosages and standardized extract formulations (e.g., specifying curcuminoid content rather than generic “turmeric”) to ensure reproducibility across trials.
- Biomarker validation: There is a critical need to validate sensitive biomarkers that can detect early physiological changes in response to lifestyle interventions, distinguishing them from normal age-related variations.
- Long-term comparative trials: Multi-year RCTs are required to compare the efficacy of combined interventions against standard pharmacological treatments (e.g., bisphosphonates or HRT) to determine if lifestyle changes can serve as a viable monotherapy or strictly as an adjunct.
- Dose-response studies: Few studies have investigated the optimal “minimum effective dose” of exercise intensity when combined with specific nutrient loads. Elucidating these thresholds is essential for prescribing precise, achievable lifestyle prescriptions.
